# Persistent circulation of a fluoroquinolone-resistant *Salmonella enterica* Typhi clone in the Indian subcontinent

**DOI:** 10.1093/jac/dkz435

**Published:** 2019-10-26

**Authors:** Carl D Britto, Zoe A Dyson, Sitarah Mathias, Ashish Bosco, Gordon Dougan, Sanju Jose, Savitha Nagaraj, Kathryn E Holt, Andrew J Pollard

**Affiliations:** 1 Oxford Vaccine Group, Department of Paediatrics, University of Oxford, and the NIHR Oxford Biomedical Research Centre, Oxford, OX3 7LE, UK; 2 Department of Infectious Diseases, Central Clinical School, Monash University, Melbourne, Victoria, 3004, Australia; 3 Department of Medicine, University of Cambridge, Cambridge, UK; 4 St John’s Medical College Hospital, Bengaluru, India; 5 Wellcome Trust Sanger Institute, Wellcome Genome Campus, Hinxton, UK; 6 Department of Infection Biology, Faculty of Infections and Tropical Diseases, London School of Hygiene and Tropical Medicine, London, UK

## Abstract

**Background:**

The molecular structure of circulating enteric fever pathogens was studied using hospital-based genomic surveillance in a tertiary care referral centre in South India as a first genomic surveillance study, to our knowledge, of blood culture-confirmed enteric fever in the region.

**Methods:**

Blood culture surveillance was conducted at St John’s Medical College Hospital, Bengaluru, between July 2016 and June 2017. The bacterial isolates collected were linked to demographic variables of patients and subjected to WGS. The resulting pathogen genomic data were also globally contextualized to gauge possible phylogeographical patterns.

**Results:**

Hospital-based genomic surveillance for enteric fever in Bengaluru, India, identified 101 *Salmonella enterica* Typhi and 14 *S*. Paratyphi A in a 1 year period. Ninety-six percent of isolates displayed non-susceptibility to fluoroquinolones. WGS showed the dominant pathogen was *S.* Typhi genotype 4.3.1.2 (H58 lineage II). A fluoroquinolone-resistant triple-mutant clone of *S.* Typhi 4.3.1.2 previously associated with gatifloxacin treatment failure in Nepal was implicated in 18% of enteric fever cases, indicating ongoing inter-regional circulation.

**Conclusions:**

Enteric fever in South India continues to be a major public health issue and is strongly associated with antimicrobial resistance. Robust microbiological surveillance is necessary to direct appropriate treatment and preventive strategies. Of particular concern is the emergence and expansion of the highly fluoroquinolone-resistant triple-mutant *S*. Typhi clone and its ongoing inter- and intra-country transmission in South Asia, which highlights the need for regional coordination of intervention strategies, including vaccination and longer-term strategies such as improvements to support hygiene and sanitation.

## Introduction

The Indian subcontinent is a high-incidence endemic setting for enteric fever, a febrile illness caused by *Salmonella enterica* serovars Typhi (*S.* Typhi) and Paratyphi A (*S.* Paratyphi A), which on a global scale caused an estimated 14.3 million cases of febrile illness in 2017.^1^ The inter-regional circulation of antimicrobial-resistant (AMR) *S.* Typhi within the Indian subcontinent affects the impoverished and children disproportionally while limiting treatment options. A clade of fluoroquinolone-resistant *S.* Typhi 4.3.1.2 (H58 lineage II) isolates was previously detected in patients in Patan, Nepal, between 2011 and 2014. WGS of these *S.* Typhi isolates revealed SNPs in three distinct locations of the QRDR^2^^,^^3^ of genes *gyrA* (*gyrA*-S83F and *gyrA*-D87V) and *parC* (*parC*-S80I). These ‘triple mutants’ displayed MIC ≥24 mg/L and were responsible for gatifloxacin treatment failure.^4^ Global contextualization of these isolates revealed that the triple mutants appeared to have emerged in India before entering Nepal, circa 2008, where they underwent a local expansion, infecting both adults and children.^4–6^ It is not known whether these fluoroquinolone-resistant triple mutants remain prevalent in the Indian subcontinent; previous isolates from India were obtained from travellers returning to the UK from India or from sporadic patients treated at tertiary care^7^ settings without a clear understanding of the structure of the circulating pathogen population.

## Methods

In this study, hospital-based surveillance of inpatients and outpatients was conducted at St John’s Medical College Hospital, a tertiary care setting in Bengaluru, South India, which provides care for approximately 3500 patients on an outpatient basis and has a capacity of 2000 inpatient beds. It is situated in the eastern part of Bengaluru, a city that is densely populated, holding a population of over 12 million residents. This hospital in Bengaluru is situated at the confluence of three South Indian states and receives referrals from neighbouring regions in the states of Tamil Nadu and Andhra Pradesh in addition to the referrals from the home state of Karnataka. No surveillance for enteric fever exists in the local public health system, and tertiary care blood culture-positive data are the only useful surrogate for enteric fever burden.

Every microbiological specimen that was positive for a typhoidal *Salmonella* organism (confirmed by biochemical and serological means) was included in the study, and tested for antimicrobial susceptibility using the Kirby–Bauer disc diffusion method and VITEK^®^, which were reported using the CLSI interpretive criteria [inhibition zone diameters ≥31 mm and MICs ≤0.06 mg/L for ciprofloxacin and ≤0.12 mg/L for ofloxacin on VITEK^®^ 2 (bioMérieux, France) were considered susceptible].^8^ These isolates were subsequently sequenced on the Illumina HiSeq 2500 platform. Raw sequence data have been deposited in the European Nucleotide Archive under project PRJEB14050, and individual accession numbers are listed in Tables S1 and S2 (available as Supplementary data at *JAC* Online). The data were subjected to SNP and phylogenetic analyses as described previously.^6^

### Ethics

Ethics approval was obtained from the Oxford Tropical Research Ethics Committee (OxTREC 586–16) and local institutional approval from the Institutional Ethics Committee (IEC) at St John’s Research Institute (140/216) and Health Ministry’s Screening Committee, India.

## Results

Over the duration of surveillance, 19641 cultures were performed, of which 3454 were significant for non-commensal pathogenic bacteria and 3.3% of these significant blood cultures were positive for a typhoidal *Salmonella* organism. Of these, 101 were *S.* Typhi and 14 were *S.* Paratyphi A; 37 (32.2%) were isolated from children (2 months to 15 years of age) and 78 (67.8%) from adults. All were susceptible to ampicillin, co-trimoxazole, chloramphenicol, cefotaxime, ceftriaxone and azithromycin and no known AMR genes or plasmids were identified. However, 110 (96%) isolates displayed non-susceptibility to ciprofloxacin and ofloxacin (Table 1), and all except one isolate (*S.* Typhi genotype 2.2.2) were resistant to nalidixic acid. These phenotypes were mediated by a variety of QRDR mutations (Table 1), which were detected in 98% of isolates. Notably, 21 *S*. Typhi strains were QRDR triple mutants (Table 1) with ciprofloxacin MIC ≥24 mg/L.

**Table 1.** Genetic determinants of fluoroquinolone resistance in the Indian isolates

Source	*S*. Typhi (*n*=101)		*S*. Paratyphi A (*n*=14)		Total
	adults	children	adults	children	
Number of isolates	68	33	10	4	115
Fluoroquinolone non-susceptible	67 (99%)	31 (94%)	9 (90%)	3 (75%)	110 (96%)
Any QRDR mutation	67 (99%)	32 (97%)	10 (100%)	4 (100%)	113 (98%)
3 QRDR mutations (*gyrA* S83F, *gyrA* D87N, *parC* S80I)	12 (18%)	9 (27%)	0	0	21 (18%)
2 QRDR mutations	12 (18%)	4 (12%)	0	0	16 (14%)
*gyrA* S83F, *parC* E84G	6 (9%)	3 (9%)	0	0	
*gyrA* S83F, *parE* A364V	4 (6%)	1 (3%)	0	0	
*gyrA* S83Y, *parE* A364V	1 (1%)	0	0	0	
*gyrA* D87N, *parE* A364V	1 (1%)	0	0	0	
1 QRDR mutation	43 (63%)	19 (58%)	10 (100%)	4 (100%)	74 (64%)
*gyrA* S83F	7 (10%)	4 (12%)	7 (70%)	3 (75%)	
*gyrA* S83Y	34 (50%)	15 (45%)	3 (30%)	1 (25%)	
*gyrA* D87N	1 (1%)	0	0	0	
*gyrB* S464F	1 (1%)	0	0	0	

WGS data showed the enteric fever cases were caused by a diverse population of pathogens, comprising six distinct *S.* Typhi genotypes (Figure S1): 4.3.1 (*N*=89, 88%), 3.3.1 (*N*=7, 7%), 2.2.2 (*N*=2, 2%), 2.5.1 (*N*=1, 1%), 3.0.1 (*N*=1, 1%) and 3.1.1 (*N*=1, 1%). The dominant 4.3.1 (H58) genotype was further stratified into lineage I (4.3.1.1, *N*=22, 21.8%) and lineage II (4.3.1.2, *N*=67, 66.3%). All 21 QRDR triple mutants were genotype 4.3.1.2, indicating 31% frequency of fluoroquinolone resistance in this lineage. The *S*. Paratyphi A population comprised A1, C4 and C5 lineages (Figure S2).

**Figure 1.**
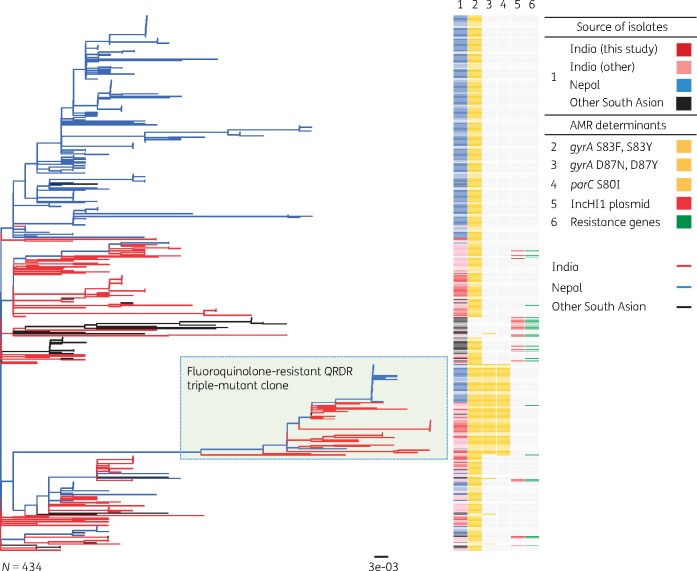
Maximum likelihood phylogenetic tree of 4.3.1.2 (H58 lineage II) *S.* Typhi isolates from Bengaluru in context with available WGS data from South Asia. The fluoroquinolone-resistant QRDR triple-mutant clone is shown in light green. Branches and rings are coloured according to the inset legend. Branch lengths are indicative of the estimated substitution rate per variable site; the tree was outgroup rooted using *S.* Paratyphi A strain AKU_12601. This figure appears in colour in the online version of *JAC* and in black and white in the print version of *JAC*.

For regional and global contextualization, whole-genome phylogenies were constructed including *S*. Typhi 4.3.1.2 from this study and from previously published global collections.^5^^,^^9^ This *S*. Typhi 4.3.1.2 tree comprised 434 isolates inferred from an alignment length of 1235 SNPs. *S*. Typhi 4.3.1.2 isolated in Bengaluru were not monophyletic in the tree; rather they formed multiple distinct clades intermingled with other isolates from India, Nepal and Bangladesh, indicating broad regional circulation. The most recent common ancestor (MRCA) of Bengaluru isolates was the MRCA of all South Asian *S*. Typhi 4.3.1.2 (Figure 1), supporting the hypothesis that this genotype emerged in India and has since been circulating in the region. The triple mutants isolated in Bengaluru formed a monophyletic clade with those observed previously in both India and Nepal^4^^,^^6^ (Figure 1), indicating a single emergence event in India followed by ongoing clonal expansion, leading to both persistence in India and regional spread.

## Discussion

In the absence of a reliable public health surveillance system, these data potentially reflect the occurrence of enteric fever in this South Indian setting and the high-resolution data obtained as a result of pathogen WGS enabled insight into the composition of the circulating population structure and contextualization of strains on a regional and global level. The close genetic relatedness of *S.* Typhi isolated in South Asia (Figure 1), including the fluoroquinolone-resistant clone, indicates inter-regional transmission and suggests that enteric fever prevention strategies require a coordinated approach between these countries. Similar findings exist with regard to the *S.* Paratyphi A population, in which strains belonging to the A1 lineage cluster with those of other countries of South Asia while lineages C4 and C5 cluster more closely with those of China and South-East Asia (Figure S2). The lack of an *S.* Paratyphi A vaccine limits prevention options.

The dominance of the 4.3.1 genotype in this study is consistent with recent reports from other endemic regions of South Asia, South-East Asia and East and Southern Africa. However further characterization of the 4.3.1 population revealed a greater dominance of lineage 4.3.1.2 compared with lineage 4.3.1.1, potentially associated with local selection from fluoroquinolone use.^10^ Notably, no MDR (i.e. combined resistance to ampicillin, co-trimoxazole and chloramphenicol)^5^^,^^6^ was observed in this study. This is in contrast with recent data from Bangladesh that identified 4.3.1.1 as a dominant genotype, which in that setting was associated with QRDR triple mutants and MDR.^11^ Cephalosporins and azithromycin are currently the first-line treatment for enteric fever in the majority of South Asian settings. No cephalosporin resistance was detected in these isolates; however, it is anticipated that this could emerge via the acquisition of plasmid-encoded ESBL genes, as has recently been observed among *S*. Typhi isolates from other parts of India and neighbouring Pakistan.^12–15^

Our study has limitations as all isolates examined were from a single hospital-based passive surveillance programme and thus may not be representative of the disease trends in the wider community. We also did not actively survey all febrile cases coming to the hospital and it is known that hospital-based active surveillance of enteric fever yields a higher number of cases.^16^ However, data from a global collection including isolates from India and from Nepal suggest the genotypes obtained from this study are fairly representative of strains circulating in the region and provide interesting insight into the molecular structure of the local pathogen population. The other limitations are related to issues intrinsic to the currently available tools used for diagnosis, sequencing and bioinformatic pipelines. These include the low sensitivity of blood culture, the use of short-read sequencing technology and the reliance on an exhaustive list of genes and SNPs to identify molecular mechanisms of AMR in these isolates.

### Conclusions

These data, we believe, constitute the first genomic surveillance study of blood culture-confirmed enteric fever in India, and provide valuable insights into the circulating pathogen population. Of particular concern is the emergence and expansion of the highly fluoroquinolone-resistant triple-mutant *S*. Typhi clone, which was responsible for 18% of all enteric fever cases in this setting. Its ongoing inter- and intra-country transmission in South Asia highlights the need for regional coordination of intervention strategies, including vaccination but also infrastructure improvements to support hygiene and sanitation.

## Supplementary Material

dkz435_Supplementary_Data
