# Supplementary material for: Persistent circulation of a fluoroquinolone-resistant Salmonella enterica Typhi clone in the Indian subcontinent
Source: J Antimicrob Chemother. 2019 Oct 26;75(2):337–41. doi: 10.1093/jac/dkz435 (PMC7223262; doi:10.1093/jac/dkz435)
Supplement: dkz435_Supplementary_Data [file dkz435_supplementary_data.zip › Figures_S1_and_S2.docx]

**Supplementary data**

**Figure S1**. **Distribution of typhoidal *Salmonella* serovars, stratified by age group.** Barplots show frequency of isolates of each pathogen subtype (species/genotypes/lineage), coloured as per the inset legend.


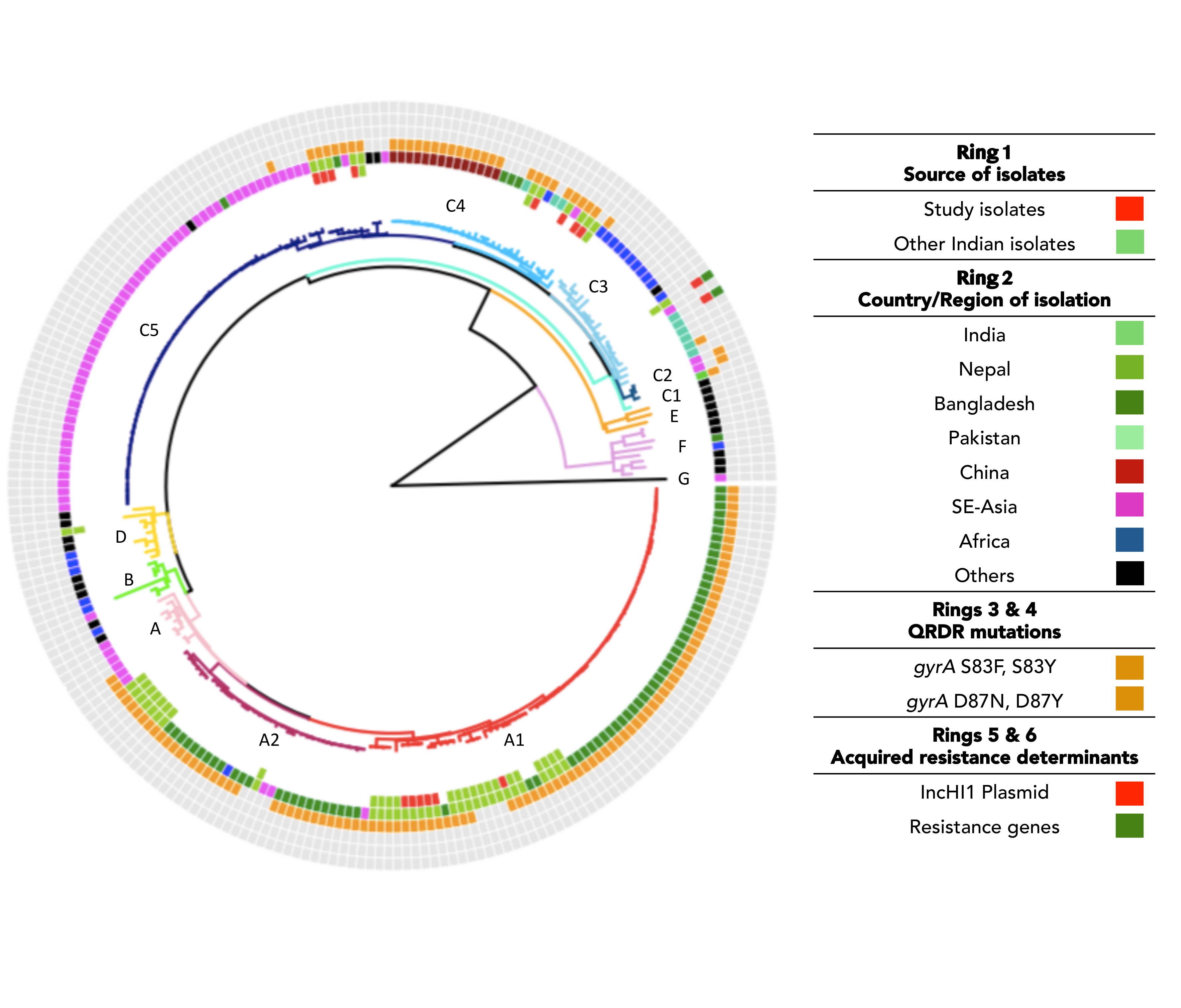


**Figure S2: Global contextualization of *S.* Paratyphi A strains isolated in this study.**

Maximum likelihood tree including the 14 novel *S*. Paratyphi A isolates from this study together with 242 global isolates (outgroup-rooted using *S.* Typhi CT18). Clades previously defined in Zhou *et al* are labelled. Rings are coloured according to the inset legend.
